# LIFELONG LEARNING AND SELF-EFFICACY OF PHYSICAL ACTIVITY ENGAGEMENT IN SEDENTARY OLDER ADULTS: A QUALITATIVE STUDY

**DOI:** 10.1093/geroni/igad104.2192

**Published:** 2023-12-21

**Authors:** Claire Wang, Mengchi Li, Jing Huang, Yeabsira Tufa, Junxin LI

**Affiliations:** Johns Hopkins University, Baltimore, Maryland, United States; Johns Hopkins, Baltimore, Maryland, United States; Johns Hopkins University, Baltimore, Maryland, United States; Johns Hopkins University, Baltimore, Maryland, United States; Johns Hopkins University, Baltimore, Maryland, United States

## Abstract

Societal-level norms play an essential role in later-life sentiments surrounding learning. While there is a wealth of literature on the benefits of learning new skills in improving health and physical capacity, there is a gap in how learning perceptions can influence willingness to learn in contemplation, action, and outcome. With sedentary lifestyles increasing, improving learning and self-efficacy will be crucial for physical activity engagement. In a randomized-controlled trial study, we explore the interface between lifelong learning and aging self-efficacy in the “third age,” where participants were asked to learn about wearable activity trackers and strengthening exercises. Twenty-nine older adults (mean age 71.1 ± 4.5, sleep problems, exercising ≤ 2x per week, sedentary activity > 6h/day) were included in semi-structured post-study interviews after participating in the 24-week intervention. Qualitative data was analyzed through NVivo, applying a grounded theory approach, while coding categories were produced inductively. Topics of diminished later-life learning and self-efficacy were tied to age-related social norms like retirement, value in age rather than wisdom, and a decline ideology of age. Ambivalence in action was another critical finding related to how participants felt conflicting emotions regarding whether they could complete or learn in the intervention (self-efficacy). However, given both opportunity and encouragement, these same participants learned new skills and thrived in the intervention. This research calls for inclusive approaches to learning that combat ageism-related societal norms and provide conducive environments for hedonic learning motivation. It provides insight into intervention adherence, capacity-building, and manifestations of internalized ageism on later-life learning outcomes.

